# A bioprinted model of pregnant human uterine myometrium

**DOI:** 10.3389/fbioe.2025.1632320

**Published:** 2025-10-30

**Authors:** Craig Ulrich, Korrina Siddiqui, Lexa K. Baldwin, Weijian Hua, Jacob K. Kuklok, Jada J. Okaikoi, Lauren L. Parker, Juli Petereit, Dave R. Quilici, Grace M. Silva, Anutr Sivakoses, Jiavanna S. Wong-Fortunato, Rebekah J. Woolsey, Adrian West, Yifei Jin, Heather Burkin

**Affiliations:** ^1^ Department of Pharmacology, University of Nevada, Reno School of Medicine, Reno, NV, United States; ^2^ Department of Mechanical Engineering, University of Nevada, Reno, NV, United States; ^3^ Nevada Bioinformatics Center, Reno, NV, United States; ^4^ Nevada Proteomics Center, Reno, NV, United States; ^5^ The University of South Florida Morsani College of Medicine, Tampa, FL, United States; ^6^ The University of Arizona Cancer Center, Tucson, AZ, United States; ^7^ The University of Nebraska Medical Center, Department of Pathology, Microbiology, and Immunology, Omaha, NE, United States; ^8^ Department of Physiology and Pathophysiology, University of Manitoba, Winnipeg, MB, Canada; ^9^ Biology of Breathing Research Theme, Children’s Hospital Research Institute of Manitoba, Winnipeg, MB, Canada; ^10^ Department of Obstetrics and Gynecology, University of Nevada, Reno School of Medicine, Reno, NV, United States

**Keywords:** uterus, labor, myometrium, pregnancy, bioprinting, tissue model

## Abstract

Despite decades of research, complications associated with dysfunctional labor are leading causes of maternal and neonatal morbidity. Currently available experimental models are not sufficient to understand the complex mechanisms underlying human labor nor to test new therapeutic approaches. We sought to develop a bioprinted tissue model of pregnant human myometrium that replicates the morphological, contractile and molecular characteristics of native pregnant human uterine myometrium as a resource to accelerate basic discovery and pharmacological testing. We have utilized primary human uterine smooth muscle cells to bioprint myometrial tissue rings containing >75% viable cells with elongated, smooth muscle morphology. Immunofluorescence confirmed expression of smooth muscle markers (caldesmon, alpha smooth muscle actin, and smooth muscle myosin), contractile-associated proteins (oxytocin receptor, prostaglandin receptors and connexin-43), and steroid hormone receptors (estrogen and progesterone receptors) characteristic of pregnant human uterine myometrium. Bioprinted tissues contracted in response to physiological agonists oxytocin (p < 0.001), prostaglandin F_2α_ (p = 0.003), and prostaglandin E2 (p < 0.001), and relaxed in response to the nitric oxide donor S-nitrosoglutathione (p = 0.004). Further development of this model could provide an abundant and homogeneous tissue source to facilitate mechanistic studies and test agents to modulate labor.

## Introduction

Human labor is characterized by the development and maintenance of coordinated contractions of the uterine smooth muscle (myometrium). The premature initiation of this process results in preterm delivery, a leading cause of neonatal morbidity and mortality (Pehlivanoglu et al., 2013; Bradley et al., 2025). Weak or irregular contractions lead to labor dystocia and the need for delivery by Caesarean section, while excessive contraction strength or frequency can result in hypoxia and fetal distress (Barber et al., 2011; Pehlivanoglu et al., 2013). Despite decades of research, the molecular pathways underlying labor are not completely understood and complications associated with labor dystocia and preterm delivery remain leading causes of Caesarean section and neonatal morbidity respectively (Osterman et al., 2021). Therefore, there is an urgent need to develop biologically relevant model systems of normal and pathological pregnancy conditions.

Several animal models have been used to study the molecular mechanisms underlying the transition of the uterus to the contractile state (Elovitz and Mrinalini, 2004; Mitchell and Taggart, 2009). Rodent models are relatively inexpensive and can be genetically manipulated; however, differences between the regulation of rodent and human parturition include gestation length, fetal numbers, placentation, progesterone regulation, and uterine anatomy, histology, and physiology (Elovitz and Mrinalini, 2004; Mitchell and Taggart, 2009; Aguilar and Mitchell, 2010; Malik et al., 2021). Large animal models (ovine and porcine) are expensive and exhibit differences in gestation length, fetal numbers, placentation, uterine anatomy and hormonal regulation (Mitchell and Taggart, 2009; Nielsen et al., 2016; Sun et al., 2023). Non-human primates serve as excellent physiological models, but their accessibility is limited by cost (Elovitz and Mrinalini, 2004; Adams Waldorf et al., 2011; Li et al., 2021a). No animals display a high prevalence of spontaneous preterm birth, and available models require interventions to induce early labor (Nielsen et al., 2016). Therefore, it is important to confirm findings from animal models in relevant human models (Mitchell and Taggart, 2009; Nielsen et al., 2016).

Another model system that has been instrumental to elucidating mechanisms underlying uterine smooth muscle function is the 2D cell culture model (Condon et al., 2002; Haluska et al., 2002; Mesiano et al., 2002). Cultured uterine myocytes have the advantages of convenience, high reproducibility, and high throughput. Uterine myometrial cells grown in culture maintain expression of key contractile proteins (Condon et al., 2002; Devost and Zingg, 2007). Cells in 2D culture cannot be used for contractile studies, with the exception of myometrial cells grown on collagen gel lattices (Devost and Zingg, 2007). Another disadvantage of 2D models is that monolayers may not accurately reflect the complex cellular interactions found in three-dimensional tissues, resulting in altered gene transcription, protein production, cytoskeletal structure, and cellular function (Ilicic et al., 2017; Souza et al., 2017; Moysidou et al., 2020). Perhaps the most physiologically relevant contractile studies have been performed with *ex vivo* human uterine myometrial tissue strips (Baumbach et al., 2012; Arrowsmith and Wray, 2014; Maxey and McCain, 2021). Human myometrial tissues are obtained from patients undergoing elective Cesarean-section and myograph-based experiments allow measurement of variables such as contraction interval, amplitude, and force. Organ tissue bath experiments allow the assessment of responses to pharmacological agents administered directly to tissues in real time; however, limitations include a limited window of tissue viability and high level of heterogeneity (Quaas et al., 1987; Maxey and McCain, 2021).

There is a need to create functionally relevant human uterine tissue models to accelerate basic pregnancy research and to reduce the need for animal testing (Park et al., 2024; Wheeler and Leach, 2025). Three-dimensional (3D) engineered tissue models have emerged as powerful complimentary tools to study contraction dynamics. Bioengineered models of normal and pathological tissues have been developed, including contractile models of skeletal, airway, vascular, and cardiac muscle (Liu et al., 2020; Alave Reyes-Furrer et al., 2021; Gold et al., 2021; Jung et al., 2022; Kundu et al., 2022; Osagie et al., 2022; Finkel et al., 2023; Hockney et al., 2023; Song et al., 2023; Cherukuri et al., 2024; Tung et al., 2024; Dell et al., 2025). We sought to develop a comparable bioprinted uterine myometrial tissue model that aims to replicate native pregnant human uterine myometrium.

## Materials and methods

### Tissue procurement and uterine smooth muscle cell isolation

Uterine tissue biopsies were obtained from the upper edge of the transverse incision in the lower uterine segment from women delivering via Caesarean section at Renown Regional Medical Center, Reno, NV under informed consent and with approval from the University of Nevada and Renown Regional Medical Center Institutional Review Boards (Ulrich et al., 2019; Ulrich et al., 2025). Tissue donors were pregnant individuals at 38–39 weeks of gestation in the absence of HIV or hepatitis infection. Medical history data was collected and de-identified. Patient ages ranged from 24 to 35 years (mean age 28 years). All were Caucasian, singleton pregnancies, and parity 1–5 (mean 2).

Uterine myometrium was dissected and used for myometrial cell isolation. Myometrial cells were separated using a gentleMACS dissociation instrument (Miltenyi Biotec, Bergisch Gladbach, Germany). Cells were strained, centrifuged, and resuspended in Dulbecco’s modified Eagle medium (DMEM; ThermoFisher Scientific) containing 10% fetal bovine serum and antibiotics. Growth media was supplemented with 60 nM 17β-estradiol and 600 nM progesterone to mimic third trimester plasma concentrations. Isolated cells were allowed to proliferate in a humidified incubator at 37 °C, 5% CO_2_.

### Rheology

A rheometer (MCR 92, Anton Paar, Ashland, VA) equipped with a cone-plate measuring system (cone angle of 1°, cone diameter of 50.0 mm, and cone-to-plate gap of 0.102 mm) was employed to evaluate the rheological properties of the prepared alginate-Matrigel composites. The steady shear rate sweeps were carried out to measure the yield stress and viscosity of the composites pre- and post-crosslinking. Specifically, the composites with 0.5, 0.6, and 0.7% (w/v) alginate were selected as the pre-crosslinking samples, while the composites with 0.5% and 0.6% (w/v) alginate were used as the post-crosslinking samples. In the measurements, the shear rate was increased from 10^–2^ to 10^–3^ s^-1^ and the shear stress and viscosity at each shear rate were recorded. For the yield stress, the obtained shear stress-shear rate plots were fit into the Herschel-Bulkley model (Zhu et al., 2005). All measurements were conducted at room temperature.

### Bioink preparation

**FIGURE 1:**
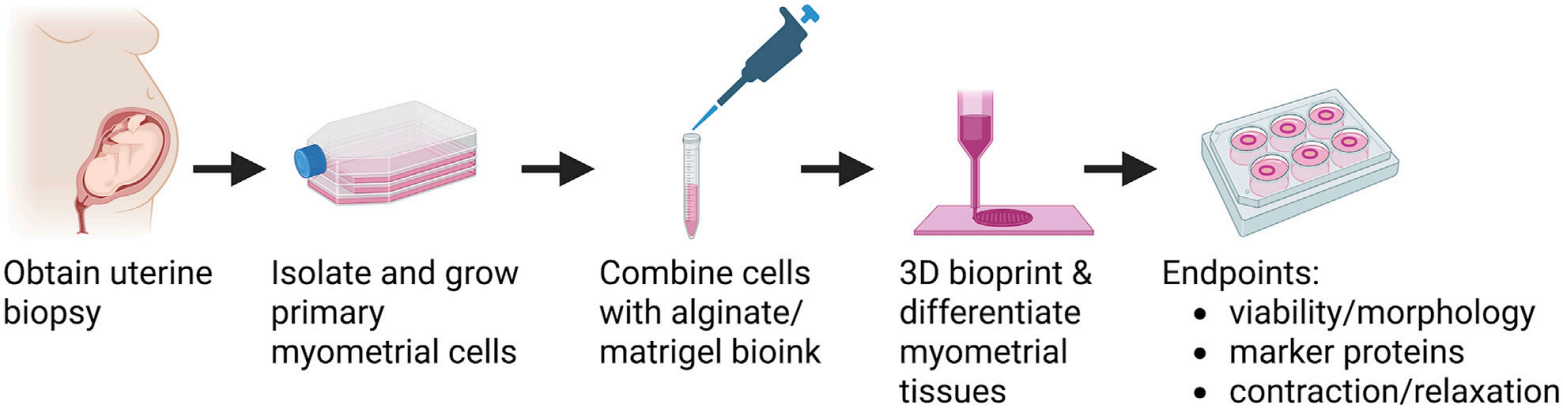
Overview of bioprinted human myometrial tissue production. Human uterine myometrial cells were isolated from uterine biopsies taken from women undergoing elective Caesarian section at term. Myometrial cells were isolated and allowed to proliferate in 2D culture, suspended in an alginate-based Bioink, and printed into 14 mm diameter rings. Bioprinted tissues were allowed to differentiate for 7 days, treated with alginate lyase, and then allowed to differentiate an additional 7 days. At day 14, tissues were assessed for cell viability and morphology, protein expression, and function. Created in BioRender. BURKIN, H. (2025) https://BioRender.com/s4rgt9b.

An overview of the experimental design is shown in Figure 1. After 3-9 passages, primary human myometrial cells were trypsinized, collected by centrifugation, and combined with bioink for 3D printing. For initial optimization experiments, bioink contained 2.5 × 10^7^ myometrial cells/mL in 50% Matrigel (Corning) and 0.5%, 0.55%, 0.60%, 0.70%, or 0.75% (w/v) NOVATACH VLVG 4GRGDSP alginate (Novamatrix) in Dulbecco’s Phosphate-Buffered Saline (DPBS) lacking calcium and magnesium (ThermoFisher Scientific).

### 3D bioprinting parameters

Bioink was deposited onto Transwell culture inserts (Grenier Biosciences) in 14-layer, 14 mm Mean Diameter rings using an RX1 Microfluidic Bioprinter (Aspect Biosystems Ltd., Vancouver, Canada) (Dickman et al., 2020). The microfluidic device allows crosslinking directly in the printhead by combining bioink with a CaCl_2_-based crosslinker (Aspect Biosystems). The crosslinker coaxially surrounds the alginate bioink to rapidly form an insoluble hydrogel fiber (Dickman et al., 2020). Print conditions were 69 mbar pressure cell/bioink solution, 55 mbar pressure buffer (Aspect Biosystems Ltd.), 55 mbar pressure crosslinker (125 mM CaCl_2_ in 2% polyvinyl alcohol, Aspect Biosystems Ltd.), print speed 25 mm/s. For all subsequent experiments bioink containing 50% Matrigel and 0.6% RGD-alginate was used. Approximately 12 tissue rings were produced per mL bioink with each tissue ring containing approximately 2 million cells.

### Cell proliferation and differentiation

Myometrial cells were allowed to proliferate in hydrogel rings for 24 h and then the tissue rings were transferred to DMEM supplemented with 1% insulin, transferrin, and selenium (ITS, Gibco), proline [40 mg/L L-proline, 10 mg/L trans-4-hydroxy-L-proline, and 0.1 mg/L L-ascorbic acid 2-phosphate, Millipore Sigma (Thorrez et al., 2018)], 1% penicillin/streptomycin (ThermoFisher Scientific), 60 nM 17β-estradiol, and 600 nM progesterone (Sigma-Aldrich). An 8-mm diameter Pyrex cloning cylinder was placed in the center of each bioprinted ring to prevent excess spontaneous tissue area reduction. Media was changed every 2–3 days and cells within synthetic rings were allowed to differentiate and form interconnected networks at 37 °C, 5% CO_2_. After 1 week, synthetic tissue constructs were treated with 0.4 mg/mL alginate lyase in differentiation medium for 5 min at 37 °C and then placed at 4 °C for 15 min to dissolve remaining alginate (Liang et al., 2016). Tissue rings were allowed to differentiate an additional 6–7 days at 37 °C, 5% CO_2_.

### Analysis of cell viability and morphology

Cell viability and morphology assessments were performed on unfixed, intact tissue rings (live cells) 2 weeks post-printing. Tissue rings were submerged in PBS containing calcium and magnesium. Synthetic rings were incubated with 2 µM Calcein AM and 1 μg/mL Hoechst for 20 min at 37 °C. Propidium iodide was added to 1 μg/mL for an additional 5 min at 37 °C. Tissue rings were rinsed with PBS, and fluorescent images were captured on an Olympus Fluoview 1,000 confocal microscope (Olympus). In some experiments, z-stack images were captured to subjectively assess cells in 3 dimensions (Supplementary Video S1).

Cell morphology and cell viability images were analyzed using ImageJ/FIJI software. The percentage of viable cells was determined by dividing the number of cells stained with Calcein AM (living cells) by the number of cell nuclei detected with Hoechst (total cells). Cell elongation percentage was determined by comparing total number of visibly elongated cells divided by cells that were visibly circular. Native human uterine smooth muscle is characterized by high numbers of parallel elongated cells, and this morphology is expected to promote contraction.

### Immunofluorescence

Two weeks after printing, synthetic tissue constructs were submerged in 4% paraformaldehyde for 5–10 min, permeabilized in 0.5% Triton X-100 for 3 min, washed 3 × 5 min in PBS, and blocked in PBS containing 5% BSA at 4 °C overnight. Tissues were incubated with primary antibody diluted in PBS containing 1% BSA in a humid chamber at 4 °C overnight, followed by secondary antibody diluted in PBS containing 1% BSA at 4 °C overnight. A complete list of antibodies can be found in Supplementary Table S1. Tissues were incubated in 1 μg/mL Hoechst for 5 min at room temperature, followed by 3 washes with PBS. Fluorescent images were captured from intact tissue rings with an Olympus Fluoview 1,000 confocal microscope (Olympus).

### Contraction and relaxation experiments

Experiments were performed to determine if synthetic myometrial constructs would respond to physiological contractile and relaxing agents. At 14 days post printing, Pyrex cylinders were removed using tweezers and a pipette tip to gently push the tissue ring constructs off the cylinders. Rings were then allowed to equilibrate 1–2 h at 37 °C, 5% CO_2_. Tissue constructs were treated with either 10 nM oxytocin (OT), 1 µM prostaglandin F_2α_ (PGF_2α_), 1 µM prostaglandin E2 (PGE2), or 100 µM S-nitrosoglutathione (GSNO) and incubated at 37 °C, 5% CO_2_ for 2 h. Control was equal volume of PBS for all groups. Myometrial tissue constructs were photographed prior to addition of contractile (OT, PGF_2α_, and PGE2) and relaxing (GSNO) agents and 2 h post treatment. Changes in inner ring diameter were auto calculated in ImageJ FIJI and the percent change in area was calculated in Excel. All contractile and relaxation experiments were performed 2–3 times with 3-6 technical replicates per treatment.

### Statistical analyses

Treatments were coded and samples randomized so those collecting and analyzing data were blinded to the treatment group (Landis et al., 2012). Experiments were performed with 3-6 technical replicates per group. Differences between groups were determined using unpaired, two-tailed t-tests or One-way ANOVA with Tukey’s multiple comparisons test.

## Results

### Rheological measurements

**FIGURE 2:**
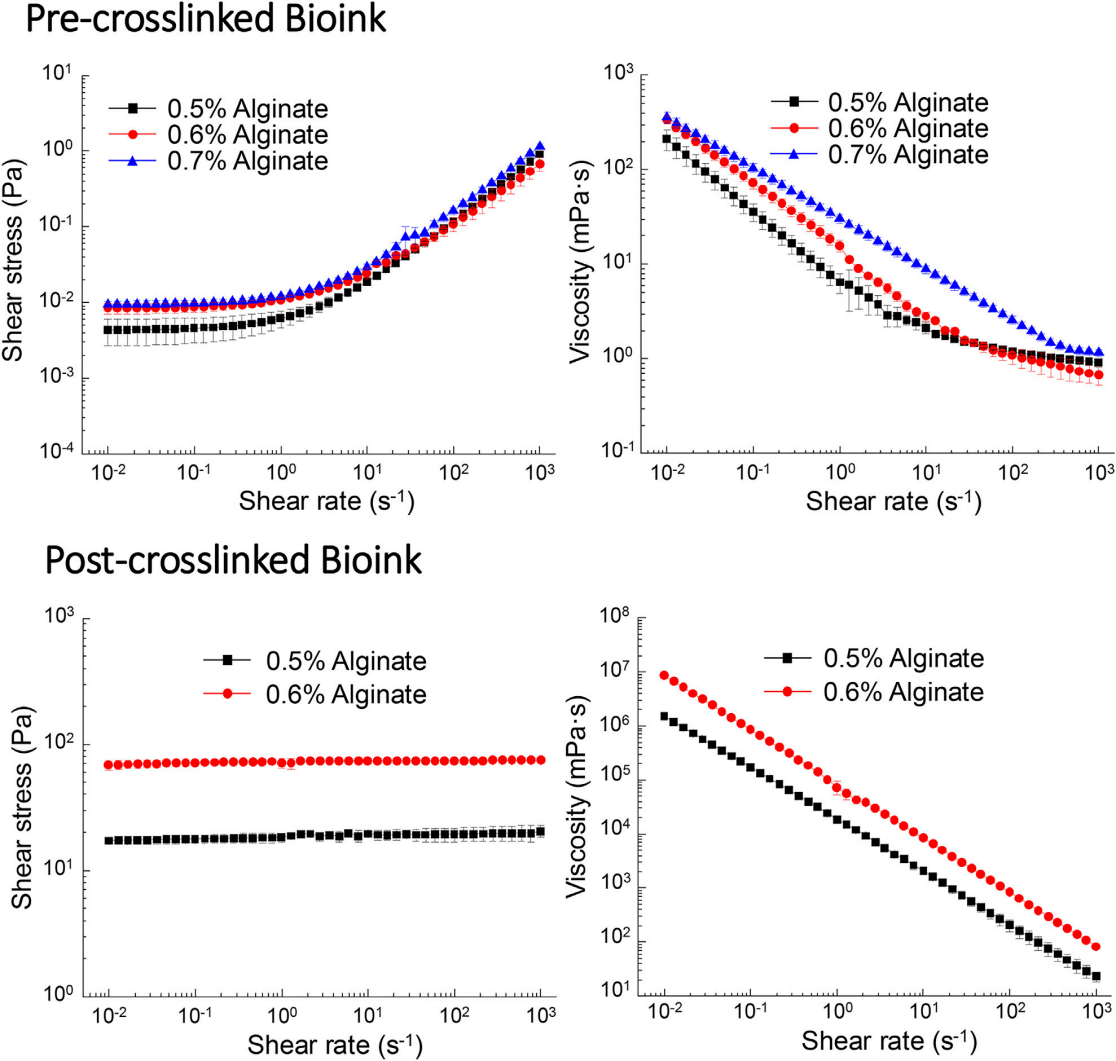
Shear stress and shear viscosity of the bioink are dependent on alginate concentration. Pre-crosslinked bioink (0.5% and 0.6% alginate) exhibited shear thinning, while post-crosslinked bioinks (0.5% and 0.6%) displayed characteristics of Newtonian fluid. In contrast, bioink containing 0.7% alginate displayed Newtonian characteristics prior to crosslinking and could not be assessed post-crosslinking.

To investigate the effects of alginate concentration on the printability of alginate-Matrigel composites, the yield stress (τ_0_) (Hua et al., 2022) and dynamic viscosity (Hua et al., 2023) were selected as the primary rheological parameters. The measurements are illustrated in Figure 2. For the pre-crosslinking composites, all the samples exhibited a weak yield-stress property. With the increase of alginate concentration from 0.5% to 0.6% and 0.7% (w/v), the yield stress increased slightly from 0.004 to 0.008 and 0.010 Pa (Figure 2). The weak yield stress is attributed to the self-assembled protein network of Matrigel, which comprises a complex mixture of proteins, primarily laminin, collagen IV, entactin, and other extracellular matrix components (Kane et al., 2018; Flores-Torres et al., 2021), enabling the composite to behave solid-like at lower shear stresses. As shown in Figure 2, the viscosity continuously decreased with the increasing shear rate, demonstrating a typical shear-thinning behavior of the composites that facilitates the ink extrusion during bioprinting (Liu et al., 2017; Amorim et al., 2021). After crosslinking by CaCl_2_, the yield stress of the alginate-Matrigel composites was significantly enhanced. As illustrated in Figure 2, the composites with 0.5% and 0.6% (w/v) alginate have the yield stresses of 17.19 and 68.23 Pa, respectively, upon crosslinking, which can be explained by the formation of a densified 3D networked microstructure from alginate chains. Simultaneously, the crosslinked composites still possessed the shear-thinning behavior, as shown in Figure 2, indicating that these composites are still extrudable if a higher shear stress is applied.

### Optimization of cell viability and morphology

**FIGURE 3:**
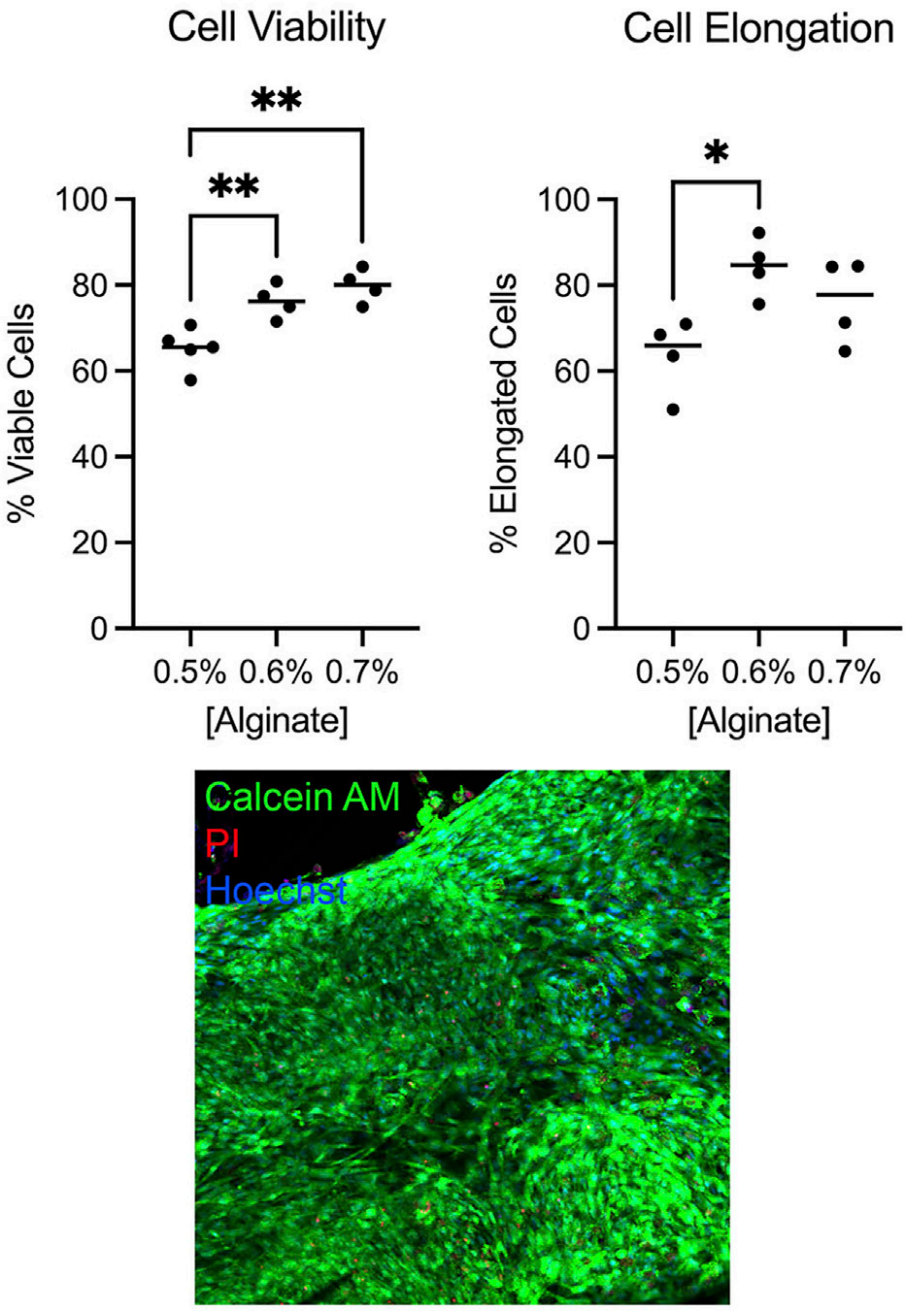
Myometrial cell viability and morphology were optimal in bioink containing 0.6% alginate. Cell viability and percent elongation in tissues printed containing varying alginate concentrations were assessed by live-dead stain with Calcein AM (live cells), propidium iodide (PI, dead cell nuclei), and Hoechst (all cell nuclei). Bioink containing 0.6% alginate yielded higher percentages of viable (**p < 0.01) and elongated (*p < 0.05) cells compared to bioink containing 0.5% alginate. Bioink containing 0.7% alginate improved cell viability (**p < 0.01) but did not significantly improve cell elongation. Individual dots represent technical replicate values. Image was captured at 20x magnification.

We determined the bioink conditions for optimal human uterine smooth muscle cell viability and morphology. Our early observations suggested bioink containing 50% Matrigel produced tissues with higher cell viability and elongation compared to bioink containing lower Matrigel concentrations or single ECM components (e.g., collagen or fibronectin alone, data not shown), consistent with the observation that high Matrigel concentrations promoted elongated morphology in bioprinted skeletal muscle (Hinds et al., 2011). RGD alginate was selected for its beneficial effects on cell adhesion and differentiation (Gribova et al., 2013; Gallagher et al., 2020). Cell viability was 65.2% when bioink was prepared with 0.5% alginate and increased to 76.2% with 0.6% alginate (p = 0.0082) and 79.8% with 0.7% alginate (p = 0.0012). The percentage of elongated cells increased from 63.5% when bioink contained 0.5% alginate to 84.3% with 0.6% alginate. We determined that bioink containing 50% Matrigel and 0.6% RGD-alginate provided the highest levels of elongated cells while retaining high numbers of viable cells (Figure 3).

### Expression of appropriate tissue markers

**FIGURE 4:**
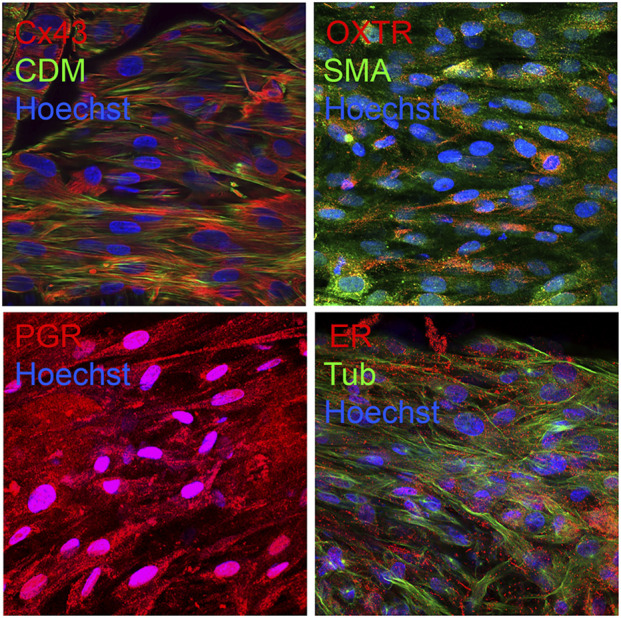
Bioprinted myometrial tissues appropriately expresses uterine and smooth muscle markers. Confocal microscopy revealed appropriate expression of contractile-associated proteins caldesmon (CDM) and smooth muscle actin (SMA), contractile-associated proteins connexin-43 (Cx43) and oxytocin receptor (OXTR), progesterone receptor (PR), and estrogen receptor alpha (ER) in bioprinted tissues. Nuclei were labeled blue with Hoechst. Images were captured at 60x magnification.

Immunofluorescence experiments were performed to confirm the expression of smooth muscle, uterine, and contractile associated proteins in bioprinted myometrial tissue constructs. We observed appropriate expression and localization of smooth muscle markers (caldesmon, α smooth muscle actin, and smooth muscle myosin), contractile-associated proteins (oxytocin receptor, prostaglandin receptors and connexin-43), and steroid hormone receptors (estrogen and progesterone receptors) in synthetic tissue constructs (Figure 4) (Chow and Lye, 1994; How et al., 1995; Rezapour et al., 1996; Myatt and Lye, 2004; de Arruda et al., 2013; Kajuluri et al., 2020; Hanuman et al., 2023).

### Synthetic human myometrial tissues respond to contractile agonists

**FIGURE 5:**
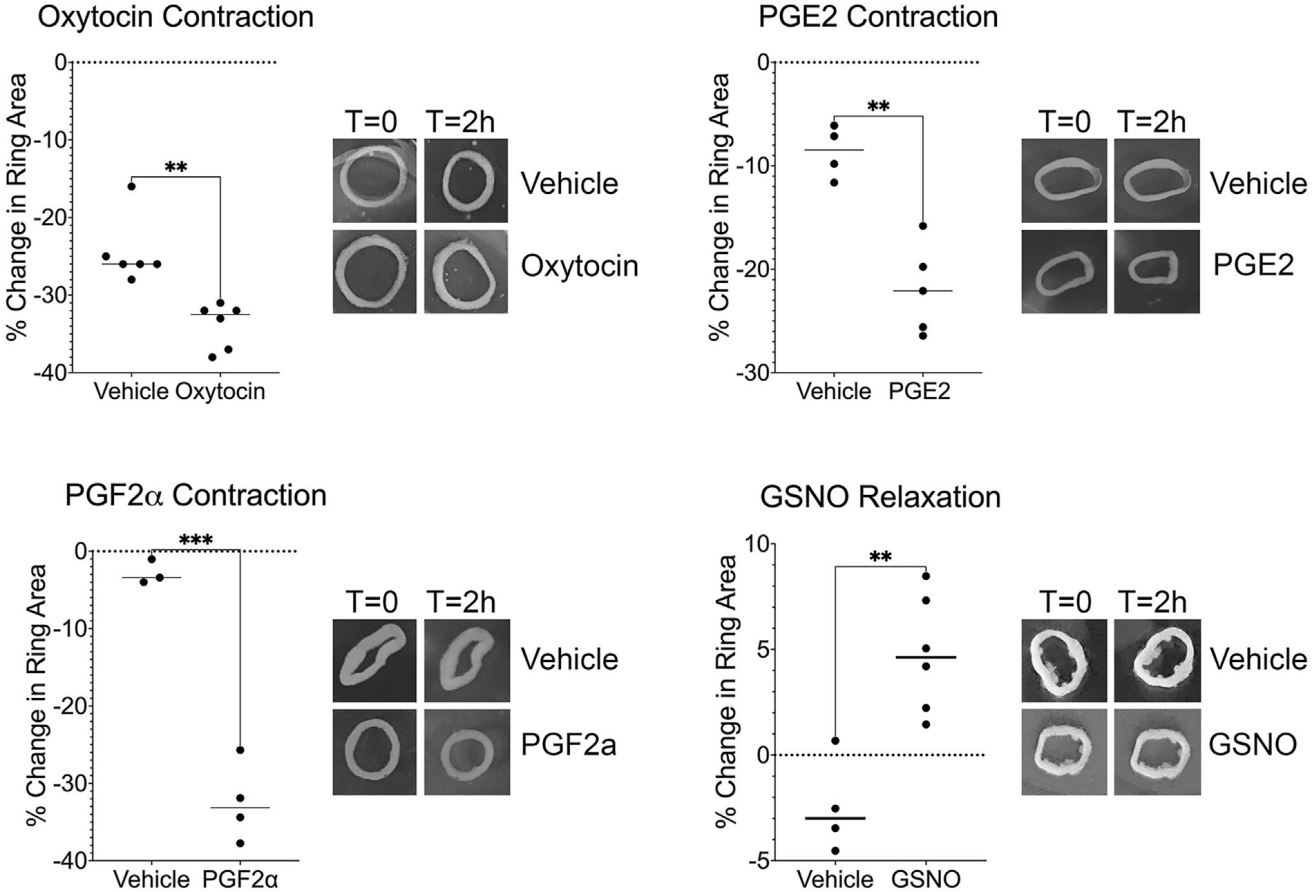
Bioprinted synthetic myometrial tissues display contraction and relaxation responses. Treatment with oxytocin, prostaglandin E2 (PGE2), and prostaglandin F_2α_ (PGF_2α_) resulted in tissue contraction as determined by a reduction in the inner ring area compared to vehicle treatment (**p < 0.01, ***p < 0.001). In contrast, tissues treated with a nitric oxide donor S-nitrosoglutathione (GSNO) showed significant relaxation as determined by an increase in ring area compared to vehicle treatment (**p < 0.01). Each technical replicate is indicated by a dot. Each graph is representative of an experiment that was repeated with 2-3 sets of bioprinted tissues. Representative tissue ring images are shown in the inserts to the right of each graph.

We detected oxytocin receptor expression in the bioprinted myometrial tissue constructs (Figure 4) and observed a 34% reduction in tissue ring area in response to 10 nM oxytocin (compared to 25% in response to vehicle; p = 0.001, Figure 5). These observations support the hypothesis that myocytes within the synthetic tissues can produce a physiological response to the contractile agonist oxytocin (Wray and Arrowsmith, 2021).

The bioprinted myometrial tissues expressed COX-2 and prostaglandin receptors (data not shown) and contracted in response to both PGE2 and PGF_2α_ (Figure 5). Tissue ring area was reduced 52.7% in response to PGF_2α_, compared to 21.5% for the vehicle controls (p = 0.002). Similarly, treatment with PGE2 reduced bioprinted tissue ring area 21.9% compared to 8.7% in vehicle-treated controls (p = 0.001). These data are consistent with observations that these inflammatory mediators promote myometrial contractions during labor (Li et al., 2021b). Together these results indicate the bioprinted myometrial tissue constructs displayed a contractile response to prostaglandins as expected for human uterine smooth muscle.

### Synthetic human myometrial tissues relax in the presence of nitric oxide donor

Previous studies have demonstrated that uterine smooth muscle exhibits a relaxation response distinct from other smooth muscle tissues. Nitric oxide (NO) induces relaxation of uterine smooth muscle in a dose-dependent manner, independent of cyclic guanosine monophosphate (cGMP) pathways (Buxton et al., 2001). Bioprinted tissue ring area increased 4.8% 2 h after addition of the NO donor S-nitrosoglutathione, which was significantly different from the 2.5% reduction in area observed in vehicle-treated control tissues. Our synthetic myometrial model demonstrated relaxation in response to the nitric oxide donor (Figure 5), providing evidence that our model can respond effectively to pharmacological intervention.

## Discussion

In recent years, bioprinted models of several reproductive tissues have been developed, including ovary, placenta, uterine endometrium, and the maternal-fetal interface (Haider and Beristain, 2023). The first *in vitro* 3D model of uterine myometrium consisted of primary human uterine smooth muscle cells carrying magnetic nanoparticles that were assembled into 3D ring structures via magnetic force (Souza et al., 2017). Immediately after magnet removal, the tissue rings displayed spontaneous contractile activity, which was inhibited with two commonly used tocolytics. To date, this model has not been used to assess agonist-induced contractions; however, this study confirmed pregnant human myometrial cells retain the ability to respond to tocolytic agents in culture. More recent data have shown that oxytocin induces intracellular calcium transients in human myometrial tissues printed in polyacrylamide hydrogels (Finkel et al., 2023). Here, we report the development of the first bioprinted tissue model of pregnant human myometrium that responds to multiple physiological contractile stimulants as well as a known tissue relaxant. This model does not require cells uptake magnetic particles, which may alter multiple cellular functions (Chen and Hou, 2023). Another relative advantage of the microfluidic bioprinting platform is that multiple cell types can be deposited in layers, so this model can serve as a foundation to develop models in which interactions between the myometrium and other cell types (such as immune or epithelial cells) can be studied. The 3D culture conditions we describe are also compatible with scalable technology to measure contraction force and frequency (Smith et al., 2022).

The bioprinted ring structure was chosen because it was expected to allow diffusion of gases and molecules through the tissue while supporting 3D intercellular interactions and allowing basic contraction assays to be performed (Dickman et al., 2020). The major uterine contractile agonists of pregnancy are oxytocin and prostaglandins. Oxytocin was the first uterine contractile agonist discovered, and synthetic analogs are commonly used to induce or accelerate labor (Vigneaud et al., 2002; Uvnas-Moberg, 2024). During labor, oxytocin released from the pituitary binds to myometrial oxytocin receptors to initiate a series of protein phosphorylation events resulting in activation of the contractile machinery (Wray and Arrowsmith, 2021). In addition, locally produced oxytocin promotes prostaglandin production (Uvnas-Moberg, 2024). PGE2 is FDA approved for labor induction (Sanchez-Ramos et al., 2024) and PGF_2α_ also promotes uterine contraction (Ricciotti and FitzGerald, 2011). Bioprinted tissues were comprised of myometrial cells obtained from term pregnant human myometrium and maintained under high levels of estradiol and progesterone to mimic physiological third trimester concentrations. The bioprinted myometrial tissue model displayed measurable contractile responses to three known agonists (oxytocin, PGE2, and PGF_2α_) at concentrations previously used in *ex vivo* pregnant human myometrial tissues by our laboratory and others (Chiossi et al., 2012; Balki et al., 2014; Ulrich et al., 2019). The observed PGE2 contraction response supports the hypothesis that our model specifically replicates term pregnant human myometrium, in contrast to nonpregnant human myometrium which relaxes in response to PGE2 (Lopez Bernal et al., 1989).

The observed contraction and relaxation responses suggest the myometrial tissue model we describe can serve as a foundation for the development of complex human uterine tissue models, including those for pathological pregnancy conditions such as preterm labor and induction failure. Currently available medications to reduce preterm labor are only effective for 48 h, which is largely insufficient to prevent preterm delivery (Lamont et al., 2016) Recent data indicate macrophages promote development of the contractile phenotype and mediate preterm parturition (Gonzalez et al., 2011; Lopez et al., 2024). Addition of immune or other cell types may allow the creation of bioengineered tissues that functionally represent human preterm laboring myometrium. The microfluidic bioprinting platform allows the deposition of multiple cell types in layers, so this bioprinted model can serve as a foundation to develop models in which interactions between the myometrium and other cell types can be studied. Labor dystocia is treated with the administration of synthetic oxytocin, but patient responses are highly variable, and oxytocin supplementation does not reduce cesarean delivery rates (Bugg et al., 2013). Proposed uterine factors underlying dystocia include inadequate expression of pro-labor proteins, insufficient contractile force, and metabolic fatigue (Kissler and Hurt, 2023). Bioprinted uterine tissues could be adapted to represent preterm labor and labor dystocia to explore underlying mechanisms and to identify and test new tocolytics and uterotonics. The idea that myometrial tissue models of pathological pregnancy conditions can be developed is supported by recent reports of engineered tissue models that replicate cardiac, skeletal muscle, and gastrointestinal disease phenotypes (Workman et al., 2017; Smith et al., 2022; Tsui et al., 2022; Cherukuri et al., 2024; Min et al., 2024; Wheeler and Leach, 2025).

One limitation of this model is the need for characterization of transcript and protein expression. Previous work has shown that, after four passages in culture, uterine smooth muscle cells display altered transcript levels for key labor-associated genes compared to parent tissues, including elevated *ESR1* and *GJA1* transcript levels and reduced *OXTR* and *PGR* transcript levels, and the reduction in progesterone receptor expression was confirmed at the protein level (Georgiou et al., 2016; Ilicic et al., 2017). In these experiments, the smooth muscle cells were maintained in serum to stimulate proliferation, followed by incubation in low serum conditions overnight. While we do not report quantitative expression assays, future experiments will determine if bioprinted myometrial tissues display elevated expression of markers compared to cells in 2D culture, as reported for other 3D tissue models (Dickman et al., 2020; Alave Reyes-Furrer et al., 2021). Myometrial cells within the bioprinted tissues required culture in serum-free differentiation medium for 2 weeks to attain optimal morphology (data not shown). Other reports suggest the longer differentiation times are required for 3D tissues to develop optimal morphology and function, and this may be associated with changes in gene expression (Dickman et al., 2020; Alave Reyes-Furrer et al., 2021). Additionally, the bioprinted myometrial tissues likely contain low levels of other cell types in addition to myometrial smooth muscle cells that may contribute to expression differences and tissue function (Hanuman et al., 2023).

These bioprinted tissue constructs could provide a foundation for a variety of mechanistic studies, including “knock out” or overexpression experiments to elucidate or confirm important players in contraction and relaxation pathways. Immortalized human uterine smooth muscle cells have successfully been used for genetic manipulation experiments, including CRISPR editing to modify the endogenous oxytocin receptor gene (Fang et al., 2024). However, to our knowledge, these experiments have not been replicated in a three-dimensional tissue environment which our current model can provide. It is important to note that bioprinted myometrial tissues exhibit reduced mechanical properties compared to their native counterparts, primarily due to weak interfacial strength between printed layers, uniform filament orientation different from native muscle fiber alignment, and porous microstructure of the bioink upon crosslinking. Since the bioprinted tissues are not strong enough to withstand the tension necessary for myography experiments, measurements of contraction force, frequency, or duration cannot currently be obtained. Addition of biomaterials such as fibrin and elastin could improve tensile strength and elasticity of bioprinted myometrium (Li et al., 2024; Wang et al., 2024). Incorporation of nanofibers produced *via* electrospinning improved cell morphology and tensile strength in a scaffold-based uterine tissue model (Hanuman et al., 2024). Despite these limitations, this model can serve as a foundation for future research aimed at developing sensitive, scalable contraction and relaxation assays in bioprinted myometrium (Alave Reyes-Furrer et al., 2021; Finkel et al., 2023; Hanuman et al., 2023).

In conclusion, we have developed a uterine myometrial tissue model that phenotypically and functionally represents morphological, contractile, and molecular characteristics of term pregnant human myometrium. This work represents a foundational step toward the long-term goal of developing bioprinted uterine tissue models that represent multiple pathological etiologies of pregnancy and can serve as an accessible resource for basic scientific discovery, toxicology studies, drug screening, and preclinical testing.

## Data Availability

The datasets presented in this study can be found in online repositories. The names of the repository/repositories and accession number(s) can be found below: https://datadryad.org/stash, doi: 10.5061/dryad.gf1vhhn0m.

## Supplementary material

SUPPLEMENTARY VIDEO S1 Z-stack image of bioprinted myometrium labeled with Calcein AM (green, live cells), propidium iodide (red, dead cells), and Hoechst (blue, cell nuclei). Stacked images were captured at 20x magnification.
